# Tuberculosis Burden, Awareness, and Health-Related Quality of Life Among Healthcare Workers at a Tertiary Care Hospital in Maharashtra, India: A Cross-Sectional Study

**DOI:** 10.7759/cureus.115509

**Published:** 2026-08-31

**Authors:** Tanuja p Barve, Shreeja Nair, Gaurav Sahu, Anuja Anil, Ataah S Qureshi, Siddhi Rane

**Affiliations:** 1 Department of Respiratory Medicine, Mahatma Gandhi Mission (MGM) Medical College and Hospital, MGM Institute of Health Sciences, Navi Mumbai, IND; 2 Department of General Medicine, Mahatma Gandhi Mission (MGM) Medical College and Hospital, MGM Institute of Health Sciences, Navi Mumbai, IND

**Keywords:** healthcare workers, health-related quality of life, india, infection control, multidrug-resistant tuberculosis, occupational health, sf-36, tuberculosis

## Abstract

Introduction

Healthcare workers (HCWs) in high-burden settings carry an occupational tuberculosis (TB) risk exceeding that of the general population. Most Indian data cover only doctors, nurses, and trainees, report raw case counts rather than category-specific rates, and rarely examine burden, knowledge, and health-related quality of life (HRQoL) together. We quantified all three across the full occupational spectrum of a hospital serving as the district TB center for Raigad district, Maharashtra, the Indian state with the highest burden of multidrug-resistant TB (MDR-TB).

Methods

This was a cross-sectional, questionnaire-based study of 250 HCWs across 10 occupational categories at a tertiary care teaching hospital in Raigad district, Maharashtra, India, over two months in 2024. Three instruments were administered: an investigator-designed TB screening questionnaire, an investigator-designed TB awareness questionnaire, and the RAND 36-Item Short Form Health Survey (SF-36). The primary outcome was the point prevalence of current active TB; secondary outcomes were TB within the preceding five years, TB awareness, and SF-36 domain scores. Proportions carry Wilson score 95% confidence intervals (CIs), categorical comparisons used Fisher exact tests with unadjusted odds ratios (ORs), and SF-36 scores were compared using the Mann-Whitney U test.

Results

Nine of 250 HCWs had current active TB (point prevalence 3.6%, 95% CI 1.9-6.7), and seven (2.8%, 95% CI 1.4-5.7) reported TB within the preceding five years; one HCW met both definitions, giving 15 distinct affected individuals (6.0%, 95% CI 3.7-9.7). Current active TB was approximately 11 times the pulmonary TB prevalence of 316 per 100,000 reported by the National TB Prevalence Survey of India. Three of the 15 (20.0%) had MDR-TB. Expressed as category-specific rates, burden was concentrated in ancillary staff: housekeeping (4/23 (17.4%)) against doctors (6/147 (4.1%)). Non-clinical and support staff carried a fourfold excess relative to clinical staff (6/42, 14.3% versus 9/208, 4.3%; OR 3.69, 95% CI 1.24-10.99, p = 0.024), as did male sex (10/90, 11.1% versus 5/160, 3.1%; OR 3.88, 95% CI 1.28-11.72, p = 0.023). Good TB awareness was recorded in 181/250 (72.4%, 95% CI 66.6-77.6) but followed a steep occupational gradient, from 96.3% of physiotherapy staff and 87.8% of doctors down to 14.3% of security personnel and 8.7% of housekeeping staff (doctors versus all non-physician staff, 87.8% versus 50.5%, p < 0.001). Among participants with complete SF-36 responses, TB-affected HCWs scored lower than unaffected colleagues across every domain, significantly so in seven of eight: overall score 52.6 (standard deviation (SD) 29.8) versus 76.7 (SD 15.5), p = 0.009, with the largest deficits in role limitation due to physical health (-32.0 points), general health (-28.9), and physical functioning (-26.3).

Conclusions

TB burden among HCWs at this center was an order of magnitude above the national population estimate and was concentrated in ancillary staff rather than physicians, precisely the group with the poorest TB knowledge. Affected HCWs showed substantial quality-of-life impairment relative to unaffected colleagues. TB infection control should extend administrative, environmental, and respiratory-protection measures, and mandatory training in local languages, to housekeeping, security, technical, and clerical staff.

## Introduction

Tuberculosis (TB), caused by the *Mycobacterium tuberculosis* complex, remains one of the leading infectious causes of morbidity and mortality worldwide [1]. Although it predominantly affects the lungs, TB can disseminate to cause extrapulmonary disease. Transmission occurs through inhalation of droplet nuclei aerosolized by individuals with infectious pulmonary TB [2]. According to the World Health Organization (WHO) Global TB Report 2022, India reported 2.14 million TB cases in 2021 of 10.6 million cases globally [3], and the Global TB Report 2023 identified India as accounting for 27% of all TB cases worldwide [4]. India's own National TB Prevalence Survey, conducted between 2019 and 2021, estimated the prevalence of microbiologically confirmed pulmonary TB among adults aged 15 years and above at 316 per 100,000 population (95% confidence interval (CI) 290-342), substantially higher than modeled estimates had suggested [5].

Healthcare workers (HCWs) who care for patients with drug-sensitive TB, drug-resistant TB, and multidrug-resistant TB (MDR-TB) face a substantially higher risk of infection than the general population because of prolonged occupational exposure [6,7]. Given the status of India as a high-burden, middle-income country [8], ensuring that HCWs remain physically and mentally fit to diagnose and treat TB is central to elimination efforts. The National Tuberculosis Elimination Programme (NTEP), guided by the National Strategic Plan (2017-2025), set a target of eliminating TB in India by 2025, defined by reductions in incidence and mortality and by elimination of catastrophic costs; despite substantial progress, this target remains unmet, and efforts continue through strengthened early detection, universal treatment, and prevention [9,10].

Several studies have examined TB burden among HCWs in India and elsewhere. Kinikar et al. found that approximately 30% of medical residents and nursing students in western India had latent TB infection (LTBI) at entry, with a 27% risk of incident TB infection at 12 months; medical residents carried a 15-fold higher risk of TB disease than the general population [11]. Kumar et al., in a more recent tertiary care cohort in Bengaluru, documented a substantial risk of TB infection among hospital employees [6]. Pai et al. identified a high prevalence of LTBI together with diagnostic and treatment delays contributing to nosocomial transmission in India [12]. In a systematic review, Menzies et al. reported a median LTBI prevalence of 63% among HCWs in low- and middle-income countries (LMICs) versus 24% in high-income countries, with a median annual incidence of TB infection attributable to healthcare work of 5.8% in LMICs [13]. Joshi et al. found a pooled LTBI prevalence of 54% among HCWs in LMICs, with annual TB disease incidence ranging from 69 to 5,780 per 100,000 [14]. Shiferaw et al. reported active TB disease among both HCWs and support staff in Ethiopian health facilities [7], and Harries et al. highlighted cough hygiene, HCW screening, and Bacillus Calmette-Guérin (BCG) vaccination as practical protective measures in low-income settings [15]. Abbas Ali et al. reported TB-related stigma in 26.75% of participants in central India, with insufficient knowledge tripling the odds of stigma [16].

Three gaps persist in this literature. First, most Indian studies sample only doctors, nurses, or trainees, so the risk borne by housekeeping, laboratory, security, clerical, and canteen staff, who work in the same air, is largely undescribed. Second, occupational distribution is usually reported as the proportion of cases falling into each job category; because such counts are dominated by the relative size of each staff group, they cannot identify which group is actually at highest risk. Third, health-related quality of life (HRQoL) among TB-affected HCWs is seldom measured against an internal comparison group drawn from the same workforce, so it is rarely possible to separate the burden of disease from the burden of the job. These gaps are of particular consequence in Maharashtra, which carries the highest prevalence of drug-resistant TB of any Indian state [17].

We therefore set out to determine, in a single tertiary care hospital that also functions as a district TB center, (i) the point prevalence of current active TB among HCWs and the proportion reporting TB within the preceding five years; (ii) whether TB burden, expressed as category-specific rates, differed by occupational group, sex, or age; (iii) the level of TB awareness across all occupational categories; and (iv) HRQoL among TB-affected HCWs compared with unaffected colleagues in the same institution.

## Materials and methods

Study design and reporting

This was a single-center, cross-sectional, questionnaire-based study. Participants were assessed on one occasion only and were not followed longitudinally; accordingly, all disease frequencies are reported as prevalence and no incidence estimates are derived from these data. The study is reported in accordance with the Strengthening the Reporting of Observational Studies in Epidemiology (STROBE) statement for cross-sectional studies [18].

Setting

The study was conducted at Mahatma Gandhi Mission (MGM) Medical College and Hospital, Sector 18, Kamothe, Navi Mumbai, in Raigad district, Maharashtra, India, over a two-month period in 2024. The institution functions as the designated district TB center for Raigad district and registers approximately 20-25 new MDR-TB cases each month, in addition to its drug-sensitive TB caseload. Maharashtra reports the highest prevalence of drug-resistant pulmonary TB of any Indian state [17]. The hospital employs staff across clinical, laboratory, administrative, and ancillary services, all of whom share common corridors, outpatient areas, and inpatient wards.

Participants

Eligible participants were HCWs aged 18-70 years employed at the study institution, defined as any individual employed in the healthcare setting, including doctors, resident doctors, interns, nurses, physiotherapists, pharmacists, laboratory and imaging technicians, housekeeping staff, security personnel, administrative clerks, and canteen employees [14]. HCWs who did not provide written informed consent were excluded. Participants were approached across clinical, laboratory, administrative, and ancillary services during working hours, and 250 consented and completed the study instruments. The number of HCWs approached and the institutional staffing frame were not recorded at the time of the study, so a response rate cannot be calculated; the implications of this study are addressed in the Limitations.

Occupational classification

Occupational role was recorded as free text. Responses were classified into 10 categories by a keyword rule applied uniformly to all records. Interns, house officers, junior residents, and senior residents were classified as doctors, since all are medically qualified or in supervised medical training and undertake direct patient care. Physiotherapy interns were classified with physiotherapy staff. Locally used designations for cleaning and portering staff, including "mama," "mausi," and "mavshi," were classified as housekeeping. Five participants who described their role only as "student," without a specified clinical attachment, could not be assigned to a service category and are reported as a separate group rather than reallocated or removed; one further response could not be classified from the text given. Both groups are retained in all denominators, and because neither contained a TB case, their inclusion is conservative with respect to the occupational comparisons reported below.

Sample size

The sample size was calculated using the formula $n=Z^{2}p\left( 1-p \right)/d^{2}$, assuming an expected prevalence (p) of 30% based on the LTBI prevalence reported among medical residents and nursing students in western India by Kinikar et al. [11], with a 95% confidence level (Z = 1.96) and an absolute allowable error (d) of 6%. This yielded 224 participants. After allowing for a 10% non-response rate, the target was rounded to 250 participants.

Case definitions

To avoid ambiguity in the reporting of disease frequency, the following definitions were applied: Current active TB: an HCW recorded in the screening instrument as a current TB patient at the time of survey, based on a clinician diagnosis reported by the participant; TB within the preceding five years: an HCW reporting a clinician-diagnosed episode of TB that commenced within the five years preceding the survey; any current or past TB: an HCW meeting either definition. Because one participant met both, the number of distinct affected individuals (15) is less than the arithmetic sum of the two categories (16), and all rates in this paper use the distinct-individual denominator.

Site of disease was classified as pulmonary, pleural, lymph node, spinal, brain, or hepatic. Where both pulmonary and extrapulmonary involvement were recorded, the case was classified as pulmonary. Drug susceptibility was recorded as drug-sensitive or multidrug-resistant, as reported by the participant based on the treating clinician's diagnosis.

The primary outcome was the point prevalence of current active TB. TB within the preceding five years and the combined any-TB measure were reported as secondary outcomes. No incidence measure was estimated.

Data collection instruments

Three instruments were administered in a single sitting. The TB screening questionnaire (Appendix A) was designed by the investigators to identify current or previous TB and to capture symptom profile, comorbidity, treatment history, adverse effects, diagnostic modality, BCG status, and experience of stigma and support during treatment. The TB awareness questionnaire (Appendix B) comprised 16 items covering the nature and etiology of TB, mode of spread, symptoms, curability, free treatment under the national program, Directly Observed Treatment, Short-course (DOTS)/NTEP, TB categories, first- and second-line drug names, the department to approach for screening, treatment duration for drug-sensitive and drug-resistant disease, adverse effects, method of diagnosis, and awareness of the Xpert MTB/RIF (Cartridge-Based Nucleic Acid Amplification Test (CBNAAT)) assay. Responses were obtained for 15 of the 16 items; the item on etiology was not completed by any participant and was excluded from scoring.

Both investigator-designed instruments were developed from NTEP guidance and published TB awareness literature, and were reviewed for content and clarity by a panel of specialists in respiratory medicine, microbiology, and community medicine before use. A formal content validity index was not computed, and internal consistency was not assessed; both instruments are reproduced in full in the appendices so that they may be independently scored and reused.

The RAND 36-Item Short Form Health Survey (SF-36) [19] was used to assess HRQoL. The instrument is freely available for academic use through RAND Health Care without a licensing fee, and was scored strictly according to RAND scoring instructions: item responses were recoded to a 0-100 scale and averaged within each domain, with higher scores indicating better health. Eight domains are reported: physical functioning, role limitation due to physical health, role limitation due to emotional problems, energy/fatigue, emotional well-being, social functioning, pain, and general health, together with an overall score averaged across six domains.

Awareness scoring

Each awareness item was scored as yes = 1, somewhat or "I think so" = 0.5, and no = 0, giving a total across the 15 completed items with a possible range of 0-15. Participants were categorized by the investigators as having good, partial, or minimal awareness upon review of the completed instrument. To confirm that these categories separated on the underlying responses, mean total scores were computed within each category and were 14.44, 10.63, and 3.49 for good, partial, and minimal awareness, respectively. The categorization was applied uniformly to all occupational groups, and both the assigned category and the continuous item score are reported.

Data handling

Data were entered into a spreadsheet at the time of collection. During preparation for analysis, three cells in the site and symptom fields were found to have been converted by the spreadsheet application into date serial values; because this conversion is deterministic, it was reversed arithmetically, recovering one site entry as combined pulmonary and pleural involvement and two symptom entries as fever with unexplained weight loss. No other values were altered, and no missing value was imputed or estimated at any point in the analysis. Where a field was incomplete, the observed denominator is stated explicitly in Results.

Statistical analysis

Data were analyzed using IBM SPSS Statistics for Windows, Version 26.0 (IBM Corp., Armonk, NY, USA). Categorical variables are summarized as frequencies and percentages with Wilson score 95% CIs, which perform better than the normal approximation for small proportions and for proportions near the boundaries. Continuous variables are summarized as mean with standard deviation (SD), and as median with interquartile range where distributions were skewed.

TB frequency by occupational category is reported as a category-specific rate, that is, the number of affected HCWs in a category divided by the number of participants surveyed in that category, rather than as the share of total cases contributed by that category. Between-group comparisons used the Fisher exact test in preference to the chi-squared test, because expected cell counts were below five in most occupational strata. Unadjusted odds ratios (ORs) with Wald 95% CIs are presented for the pre-specified comparisons of interest.

SF-36 domain scores were compared between TB-affected and unaffected participants using the Mann-Whitney U test with correction for ties, since domain scores were bounded and markedly skewed. This comparison was restricted to participants with a complete set of SF-36 item responses.

Multivariable logistic regression was not undertaken. With only 15 events, the number of events per candidate variable falls well below the conventional minimum of 10, and any adjusted model would be unstable and prone to sparse-data bias; unadjusted associations are therefore presented and are interpreted as hypothesis-generating rather than confirmatory. All tests were two-sided, and a p-value below 0.05 was considered statistically significant. Given the number of exploratory comparisons and the small number of events, p-values are presented without adjustment for multiplicity and are interpreted descriptively.

## Results

Participant characteristics

A total of 250 HCWs participated. Of these, 160 (64.0%) were female, and 90 (36.0%) were male. Mean age was 28.0 years (SD 8.4), median 24 years, range 18-58 years, and 37 participants (14.8%) were aged 40 years or above. Doctors, including interns and residents, formed the largest occupational group. The full distribution is presented in Table 1 and illustrated in Figure 1.

**Table 1:** Baseline characteristics of the study population (N = 250). Values are n (%) unless otherwise stated. Occupational categories were derived from free-text responses using the classification rule described in Materials and Methods. SD: standard deviation

Characteristic	n (%)
Sex	
Female	160 (64.0)
Male	90 (36.0)
Age (years)	
Mean (SD)	28.0 (8.4)
Median (range)	24 (18-58)
40 years and above	37 (14.8)
Occupational category	
Doctors (including interns and residents)	147 (58.8)
Physiotherapy staff and interns	27 (10.8)
Nursing staff	25 (10.0)
Housekeeping staff	23 (9.2)
Administrative and clerical staff	7 (2.8)
Security personnel	7 (2.8)
Students (occupational role not specified)	5 (2.0)
Laboratory and imaging technicians	4 (1.6)
Pharmacy staff	3 (1.2)
Canteen employees	1 (0.4)
Not classifiable from the response given	1 (0.4)

**Figure 1:**
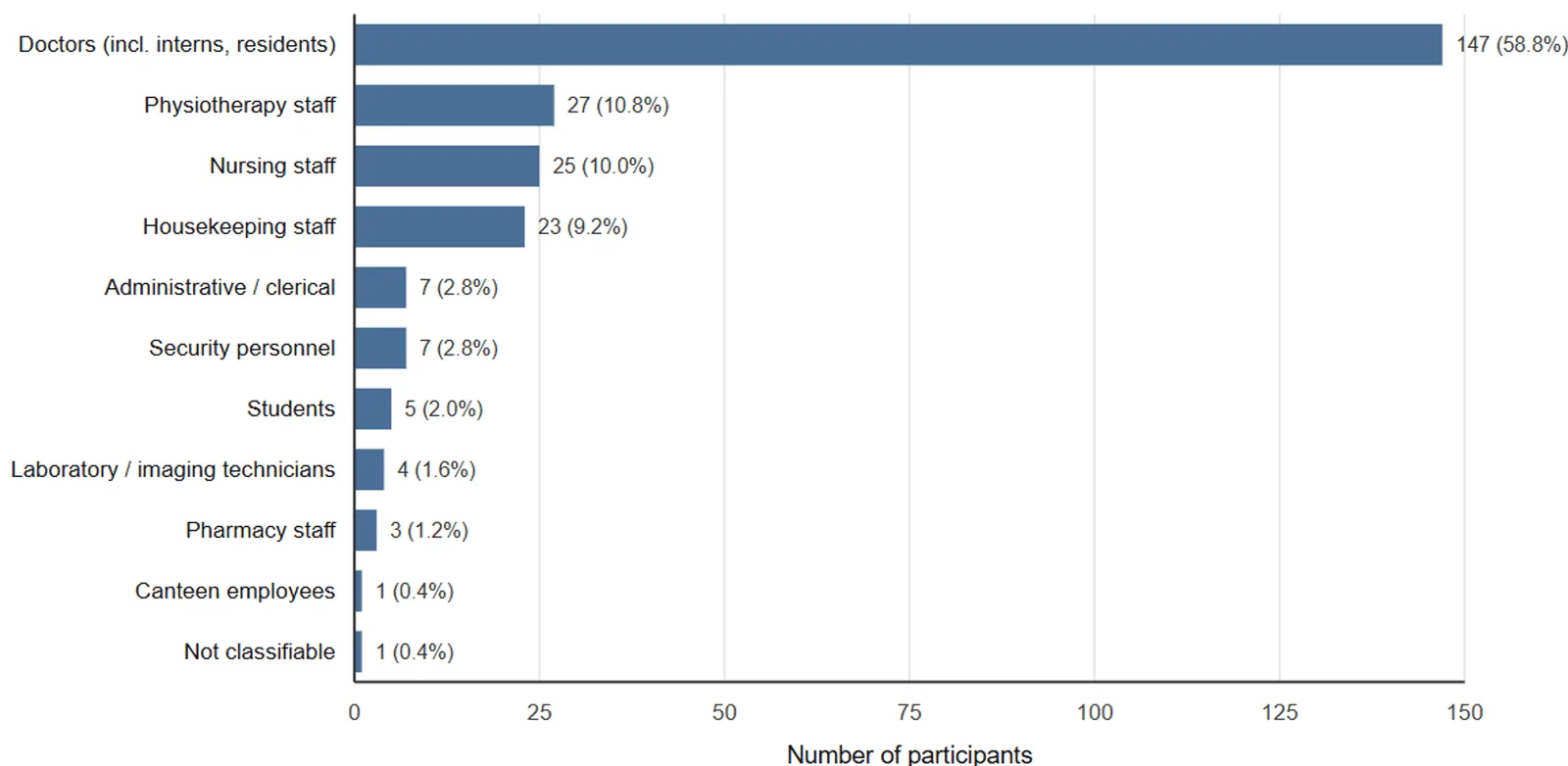
Distribution of healthcare worker categories participating in the study (N = 250).

TB burden

Nine of 250 HCWs had current active TB, giving a point prevalence of 3.6% (95% CI 1.9-6.7). Seven HCWs (2.8%, 95% CI 1.4-5.7) reported TB commencing within the preceding five years. One participant met both definitions, so the number of distinct individuals with current or recent TB was 15 (6.0%, 95% CI 3.7-9.7). Two further participants reported an episode of TB more than five years before the survey and are not included in either figure.

For context, the National TB Prevalence Survey of India (2019-2021) estimated the prevalence of microbiologically confirmed pulmonary TB among persons aged 15 years and above at 316 per 100,000 population, or 0.32% [5]. The point prevalence of current active TB in this HCW cohort was therefore approximately 11 times the national adult population estimate, and the combined current-or-recent figure approximately 19 times that estimate.

Aggregate characteristics of the 15 affected HCWs are shown in Table 2. Ten (66.7%) were male, and the mean age was 34.8 years (SD 10.5). Pulmonary disease was recorded in 11 (73.3%) and extrapulmonary disease in four (26.7%), comprising two lymph node, one pleural, and one spinal case. Three of 15 (20.0%) had MDR-TB. No affected HCW reported human immunodeficiency virus (HIV) infection or diabetes mellitus of more than one year's duration, and one (6.7%) was receiving long-term immunosuppressant therapy.

**Table 2:** Aggregate characteristics of the 15 healthcare workers with current or past tuberculosis. Values are n (%) of 15 unless otherwise stated. Percentages for tuberculosis status sum to more than 100 because one participant met both the current and the past-five-year definition. Data are presented in aggregate rather than as an individual line listing to preserve the confidentiality of a small number of identifiable staff members at a single institution. TB: tuberculosis; SD: standard deviation; HIV: human immunodeficiency virus; CI: confidence interval

Characteristic	n (%) of 15
TB status	
Current active TB	9 (60.0)
TB within the preceding 5 years	7 (46.7)
Both current and within the preceding 5 years	1 (6.7)
Sex	
Male	10 (66.7)
Female	5 (33.3)
Age (years)	
Mean (SD)	34.8 (10.5)
Range	23-55
Occupational category	
Doctor	6 (40.0)
Housekeeping staff	4 (26.7)
Nursing staff	2 (13.3)
Physiotherapy staff	1 (6.7)
Laboratory and imaging technician	1 (6.7)
Administrative and clerical staff	1 (6.7)
Site of disease	
Pulmonary	11 (73.3)
Lymph node	2 (13.3)
Pleural	1 (6.7)
Spinal	1 (6.7)
Drug susceptibility	
Drug-sensitive	11 (73.3)
Multidrug-resistant	3 (20.0)
Not recorded	1 (6.7)
Other recorded risk factors	
HIV infection	0 (0.0)
Diabetes mellitus of more than one year	0 (0.0)
Long-term immunosuppressant therapy	1 (6.7)

Symptom detail was recorded for eight of the 15 affected HCWs and was left blank for the remaining seven. Among the eight with recorded responses, unexplained weight loss was reported by all eight, fever by seven, loss of appetite by five, cough of more than two weeks and hemoptysis by four each, night sweats by three, and abdominal pain by one. Because symptom status is unknown for almost half the affected group, these proportions describe only the subgroup in which they were recorded, and no inference is drawn from them.

TB by occupational category

Case counts by occupation are strongly influenced by the size of each staff group and are therefore reported as category-specific rates (Table 3). Doctors contributed the largest absolute number of cases (six of 15), but because they also constituted the largest occupational group at 147 participants, their category-specific rate of 4.1% (95% CI 1.9-8.6) was among the lowest of any category. Housekeeping staff, at 23 participants, had a rate of 17.4% (95% CI 7.0-37.1), more than four times that of doctors. Laboratory and imaging technicians (1/4) and administrative staff (1/7) also showed high rates, although with very small denominators and correspondingly wide intervals. Figure 2 contrasts the two ways of expressing the same data and shows that the ranking reverses entirely.

**Table 3:** TB by occupational category, expressed as category-specific rates with Wilson score 95% CIs (N = 250). The final column counts distinct individuals, so it is not the sum of the two preceding columns where a participant met both definitions. CI: confidence interval; TB: tuberculosis

Occupational category	Surveyed (n)	Current active TB (n)	TB within preceding 5 years (n)	Any current or past TB, n (%, 95% CI)
Laboratory and imaging technicians	4	1	0	1 (25.0, 4.6-69.9)
Housekeeping staff	23	2	2	4 (17.4, 7.0-37.1)
Administrative and clerical staff	7	0	1	1 (14.3, 2.6-51.3)
Nursing staff	25	2	0	2 (8.0, 2.2-25.0)
Doctors	147	4	3	6 (4.1, 1.9-8.6)
Physiotherapy staff	27	0	1	1 (3.7, 0.7-18.3)
Security personnel	7	0	0	0 (0.0, 0.0-35.4)
Students	5	0	0	0 (0.0, 0.0-43.4)
Pharmacy staff	3	0	0	0 (0.0, 0.0-56.1)
Canteen employees	1	0	0	0 (0.0, 0.0-79.3)
Not classifiable	1	0	0	0 (0.0, 0.0-79.3)
Total	250	9	7	15 (6.0, 3.7-9.7)

**Figure 2:**
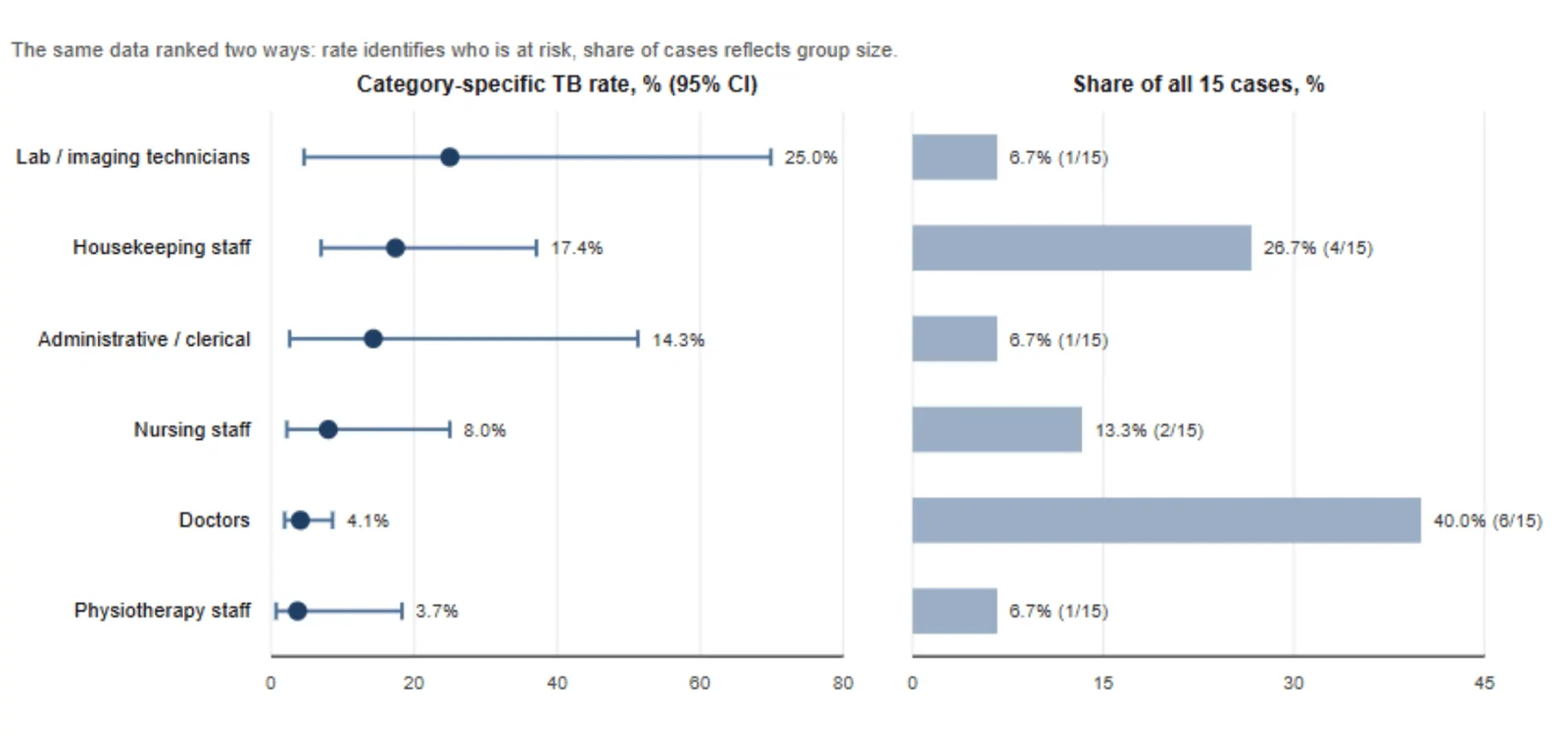
Category-specific tuberculosis (TB) rate by occupational group with 95% confidence intervals (CIs), shown alongside the share of total cases contributed by each group. The two metrics rank the occupational groups in opposite orders.

Factors associated with TB

Grouping the workforce into clinical roles (doctors, nursing, physiotherapy, and pharmacy staff) and non-clinical or support roles (housekeeping, technicians, security, administrative, and canteen staff), support staff carried more than three times the odds of TB (6/42, 14.3% versus 9/208, 4.3%; OR 3.69, 95% CI 1.24-10.99, p = 0.024). Housekeeping staff considered separately showed a similar excess (OR 4.13, 95% CI 1.20-14.24, p = 0.038). Male HCWs were more frequently affected than female HCWs (10/90, 11.1% versus 5/160, 3.1%; OR 3.88, 95% CI 1.28-11.72, p = 0.023). Age of 40 years or above was associated with TB at borderline significance (OR 3.17, 95% CI 1.02-9.88, p = 0.053). Being a doctor was not associated with increased odds of TB relative to all other categories (OR 0.44, 95% CI 0.15-1.29, p = 0.176). These associations are summarized in Table 4.

**Table 4:** Unadjusted associations with current or past TB among healthcare workers (N = 250). ORs are unadjusted with Wald 95% CIs; p-values are from the Fisher exact test. Multivariable adjustment was not performed because only 15 events were available. OR: odds ratio; CI: confidence interval; TB: tuberculosis

Factor	Exposed group, TB/n (%)	Reference group, TB/n (%)	Unadjusted OR (95% CI)	p-value
Male sex	10/90 (11.1)	5/160 (3.1)	3.88 (1.28-11.72)	0.023
Non-clinical or support role	6/42 (14.3)	9/208 (4.3)	3.69 (1.24-10.99)	0.024
Housekeeping role	4/23 (17.4)	11/227 (4.8)	4.13 (1.20-14.24)	0.038
Age 40 years or above	5/37 (13.5)	10/213 (4.7)	3.17 (1.02-9.88)	0.053
Doctor (vs. all other roles)	6/147 (4.1)	9/103 (8.7)	0.44 (0.15-1.29)	0.176

TB awareness

Good TB awareness was recorded in 181 of 250 participants (72.4%, 95% CI 66.6-77.6). Thirty-four (13.6%) demonstrated partial awareness, and 35 (14.0%) demonstrated minimal awareness (Figure 3).

**Figure 3:**
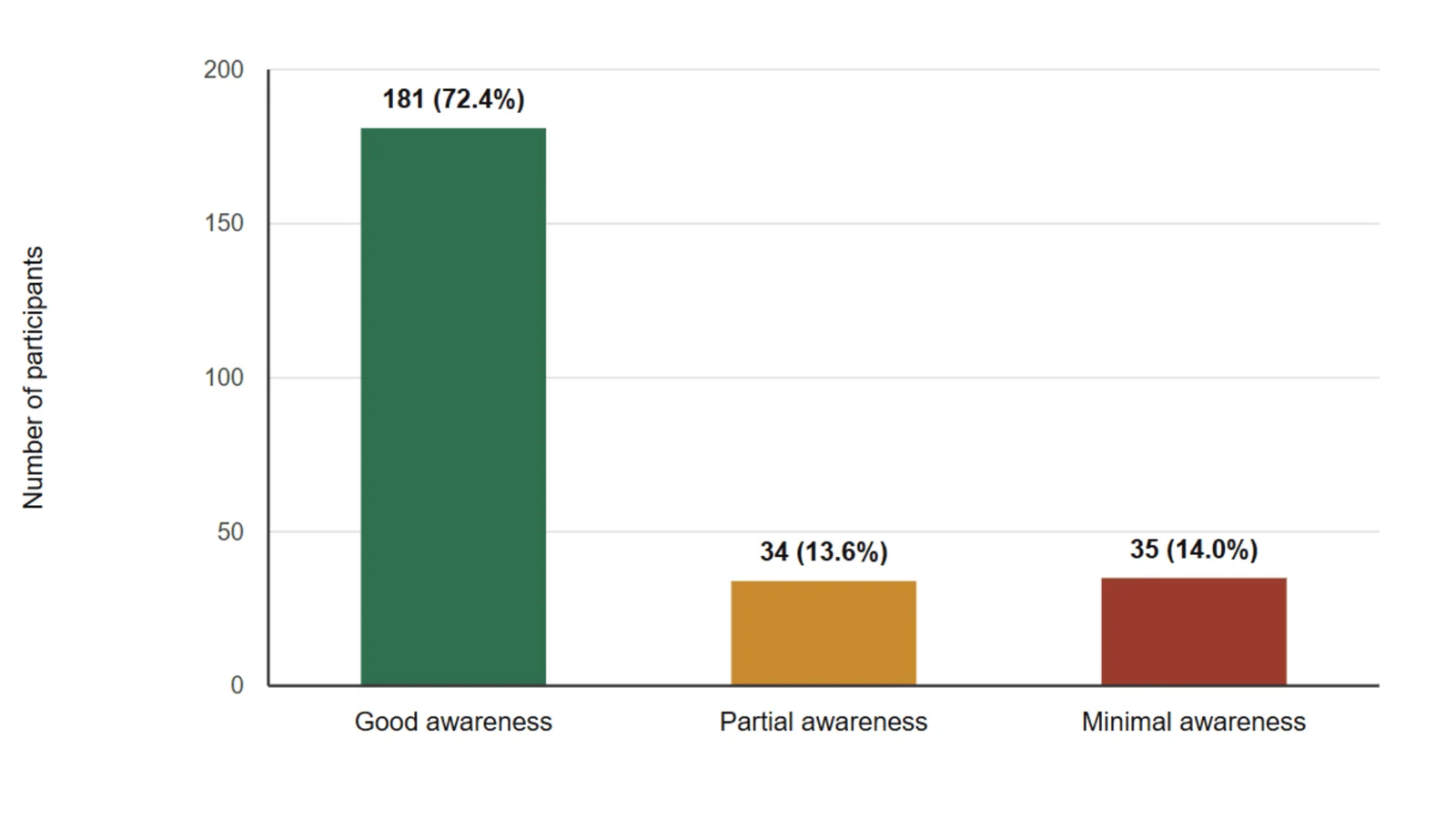
Distribution of tuberculosis awareness levels among healthcare workers (N = 250).

The aggregate figure conceals a steep occupational gradient (Table 5). Good awareness was recorded in 96.3% of physiotherapy staff and 87.8% of doctors, but in only 56.0% of nursing staff, 25.0% of technicians, 14.3% of administrative staff, 14.3% of security personnel, and 8.7% of housekeeping staff. Comparing doctors with all non-physician staff, 129/147 (87.8%) versus 52/103 (50.5%), the difference was highly significant (Fisher exact test, p < 0.001), and the same gradient was evident on the underlying item score (median 15.0 versus 12.0, Mann-Whitney p < 0.001). Awareness score did not differ between TB-affected and unaffected participants (median 15.0 in both groups, p = 0.804).

**Table 5:** Tuberculosis awareness by occupational category (N = 250). Good awareness is the investigator-assigned category; the mean item score is derived from the 15 completed awareness items scored yes = 1, somewhat = 0.5, and no = 0 (maximum 15). Categories are ordered by descending proportion with good awareness. CI: confidence interval

Occupational category	n	Good awareness, n (%, 95% CI)	Mean item score (max 15)
Physiotherapy staff	27	26 (96.3, 81.7-99.3)	14.11
Doctors	147	129 (87.8, 81.5-92.1)	13.96
Students	5	4 (80.0, 37.6-96.4)	12.80
Pharmacy staff	3	2 (66.7, 20.8-93.9)	13.00
Nursing staff	25	14 (56.0, 37.1-73.3)	12.10
Laboratory and imaging technicians	4	1 (25.0, 4.6-69.9)	7.25
Administrative and clerical staff	7	1 (14.3, 2.6-51.3)	7.86
Security personnel	7	1 (14.3, 2.6-51.3)	4.79
Housekeeping staff	23	2 (8.7, 2.4-26.8)	5.33
Canteen employees	1	0 (0.0, 0.0-79.3)	4.00
Total	250	181 (72.4, 66.6-77.6)	13.03

The occupational groups with the lowest awareness were the same groups that carried the highest TB rates. Housekeeping staff had both the second-highest category-specific TB rate (17.4%) and the lowest proportion with good awareness (8.7%).

Health-related quality of life

Complete SF-36 item responses were available for 160 participants, comprising 11 of the 15 TB-affected HCWs and 149 unaffected HCWs. TB-affected HCWs scored lower than unaffected colleagues in every domain, significantly so in seven of eight (Table 6). The largest deficits were in role limitation due to physical health (54.0 versus 85.9, a difference of 32.0 points, p = 0.014), general health (42.7 versus 71.6, -28.9, p = 0.007), role limitation due to emotional problems (57.6 versus 85.7, -28.2, p = 0.015), social functioning (52.3 versus 79.5, -27.3, p = 0.015), and physical functioning (64.5 versus 90.8, -26.3, p < 0.001). Emotional well-being was the only domain in which the difference did not reach significance (54.2 versus 71.7, -17.5, p = 0.070). The overall score across six domains was 52.6 (SD 29.8) in TB-affected HCWs against 76.7 (SD 15.5) in unaffected HCWs (p = 0.009).

**Table 6:** RAND SF-36 domain scores in TB-affected and unaffected healthcare workers with complete item responses. Higher scores indicate better health status on a 0-100 scale. p-values are from the Mann-Whitney U test with correction for ties. The overall score is the mean of physical functioning, energy/fatigue, emotional well-being, social functioning, pain, and general health. SD: standard deviation; SF-36: 36-Item Short Form Health Survey; TB: tuberculosis

SF-36 domain	TB-affected (n = 11), mean (SD)	Unaffected (n = 149), mean (SD)	Difference	p-value
Physical functioning	64.5 (28.0)	90.8 (17.3)	-26.3	<0.001
Role limitation, physical	54.0 (42.9)	85.9 (23.6)	-32.0	0.014
Role limitation, emotional	57.6 (40.9)	85.7 (24.6)	-28.2	0.015
Energy/fatigue	40.5 (31.7)	63.5 (20.9)	-23.0	0.010
Emotional well-being	54.2 (31.5)	71.7 (19.2)	-17.5	0.070
Social functioning	52.3 (38.3)	79.5 (23.1)	-27.3	0.015
Pain	61.6 (27.4)	82.8 (22.5)	-21.2	0.007
General health	42.7 (33.2)	71.6 (19.6)	-28.9	0.007
Overall score across six domains	52.6 (29.8)	76.7 (15.5)	-24.0	0.009

Median values show the same separation and indicate how far the distributions diverge: median general health was 20.0 (interquartile range 17.5-70.0) in TB-affected HCWs against 75.0 (55.0-90.0) in unaffected HCWs, and median physical functioning was 65.0 (50.0-87.5) against 100.0 (90.0-100.0). Among the 165 participants who answered the single-item general health question, 139 (84.2%, 95% CI 77.9-89.0) rated their health as good, very good, or excellent, 21 (12.7%) as fair, and five (3.0%) as poor.

## Discussion

Principal findings

In this cross-sectional study of 250 HCWs at a tertiary care hospital that also serves as a district TB center in Maharashtra, the point prevalence of current active TB was 3.6%, and 6.0% of the workforce had current or recent TB. One in five affected HCWs had MDR-TB. Three findings extend what is currently reported in the Indian literature. First, the burden was approximately an order of magnitude above the national adult population estimate for pulmonary TB. Second, when expressed as category-specific rates rather than case counts, the burden was concentrated not in doctors but in ancillary and support staff, who carried nearly four times the odds of disease. Third, the same non-clinical groups that carried the highest disease rate also had by far the poorest TB knowledge, and TB-affected HCWs showed marked impairment of quality of life across almost every SF-36 domain when compared with unaffected colleagues drawn from the same workforce.

Burden in context

Interpretation of the magnitude of this burden requires care with units. Our figure of 3.6% is a point prevalence measured on a single occasion, and it is not directly comparable with studies that report incidence per person-year. Rao et al. reported an incidence of TB of approximately 2% among resident doctors at a north Indian institution, with 11.2 new cases per 1,000 person-years in medical subspecialties [20], and Gopinath et al. reported annual pulmonary and extrapulmonary TB incidences of up to 1.80 and 1.57 per 1,000 persons, respectively, among HCWs in south India [21]. Those are rates of new disease over time; ours is a snapshot of disease present or recently past. A prevalence figure will exceed a one-year incidence figure whenever disease duration or the look-back window extends beyond a year, and our five-year look-back does exactly that. We therefore make no claim that our cohort experiences a higher rate of new infection than these earlier cohorts; the appropriate comparison is with contemporaneous population prevalence, and against the National TB Prevalence Survey estimate of 316 per 100,000 [5], the excess in this workforce is substantial and unambiguous.

For the same reason, we do not attempt to derive our observed active-TB frequency from published LTBI prevalence. Joshi et al. reported a pooled LTBI prevalence of 54% among HCWs in LMICs [14], and Menzies et al. reported a median of 63% [13], and 5%-10% of latent infections progress to active disease over a lifetime in immunocompetent hosts [22]. Multiplying these gives a lifetime cumulative risk, not a point prevalence, and the two quantities cannot be checked against one another. What the LTBI literature does establish, and what our data are consistent with, is that the reservoir of infection in this workforce is likely to be large and that active disease of the magnitude we observed is unsurprising in that context.

More recent Indian data support our findings. Kinikar et al., working in western India in the same state as our institution, documented a 27% risk of incident TB infection at 12 months among medical residents and nursing students and a 15-fold excess risk of TB disease among residents relative to the general population [11]. Kumar et al. reported a substantial risk of TB infection among employees of a Bengaluru tertiary hospital [6]. Internationally, Shiferaw et al. documented active TB among both HCWs and support staff in Ethiopia [7], and Dimitrova et al. found HCWs in Samara Oblast, Russia, to be three times more likely than the general population to develop TB, including MDR-TB [23].

Occupational distribution: why denominators matter

The most consequential result of this study emerges only when denominators are applied. Doctors contributed six of 15 cases, and a count-based reading would suggest that physicians bear the greatest occupational risk. They do not. Doctors made up 58.8% of the surveyed workforce, and their category-specific rate of 4.1% was among the lowest recorded. Housekeeping staff, at 9.2% of the workforce, had a rate of 17.4%, and non-clinical and support staff as a group carried odds of TB 3.69 times those of clinical staff (95% CI 1.24-10.99, p = 0.024). The CIs are wide, and the finding rests on six cases among support staff, so it must be treated as hypothesis-generating, but the direction is the opposite of what a count-based reading suggests, and it converges with the awareness data from the same cohort.

Several mechanisms are plausible and are, on our data, untested. Housekeeping staff clean patient rooms, sputum collection areas, and isolation rooms, often after aerosol-generating activity and often without the ventilation safeguards that apply during clinical procedures. They are typically excluded from clinical infection-control training, are less likely to be fit-tested for N95 respirators, and may have less job security with which to report symptoms or take time off for investigation. Each of these is measurable, and none was measured here. Shiferaw et al. reached a similar conclusion in Ethiopia, where support staff shared the elevated burden of their clinical colleagues [7].

None of the affected doctors worked in the Department of Respiratory Medicine. Closer adherence to personal protective equipment (PPE) among clinicians who manage TB patients daily would be one explanation, but no data on PPE availability, fit testing, or adherence were collected, and with so few cases, the observation is equally compatible with chance. It needs direct measurement before it can be treated as a finding.

Male sex was associated with nearly four times the odds of TB in this cohort, in a workforce that was 64% female. This closely mirrors the national pattern: the National TB Prevalence Survey found pulmonary TB prevalence of 472 per 100,000 in men against 154 per 100,000 in women, a ratio of approximately three to one [5]. Our observed OR of 3.88 is therefore consistent with the background sex differential in the Indian population rather than with something specific to healthcare work, and it may additionally reflect the concentration of male staff in the ancillary roles that carried the highest rates. The same survey reported higher prevalence in older age groups, which is consistent with the borderline association we observed with age of 40 years or above.

Site of disease and drug resistance

Pulmonary disease predominated, recorded in 11 of 15 cases, whereas some earlier HCW series have reported a higher proportion of extrapulmonary, particularly pleural, disease [14,20]. Pai et al. proposed that a predominance of extrapulmonary disease among HCWs may reflect progression from newly acquired primary infection rather than reactivation of latent infection [24]. Our data run in the opposite direction and so do not support that pattern in this setting. Two explanations are worth distinguishing. The first is ascertainment: pulmonary symptoms such as cough and hemoptysis are more likely to prompt sputum testing under NTEP algorithms, whereas extrapulmonary disease requires tissue sampling and may be underascertained in a questionnaire-based survey. The second is a genuine epidemiological difference, since our institution is a designated MDR-TB treatment center with a high throughput of infectious pulmonary cases and therefore an unusually high ambient exposure to aerosolized bacilli. Our design cannot distinguish these.

Three of 15 affected HCWs (20.0%) had MDR-TB. This is consistent with the position of Maharashtra as the Indian state with the highest burden of drug-resistant pulmonary TB [17] and with the role of our institution as the district TB center, registering 20-25 new MDR-TB cases monthly. HCWs in such settings are known to be at elevated risk of drug-resistant disease specifically: O'Donnell et al. documented a high incidence of hospital admissions with MDR and extensively drug-resistant TB among South African HCWs [25], and von Delft et al. described the systemic factors that leave HCWs disproportionately affected [26].

The awareness gap

Good TB awareness was recorded in 72.4% of participants, but the aggregate figure is misleading. The gradient runs from 96.3% among physiotherapy staff and 87.8% among doctors down to 14.3% among security personnel and 8.7% among housekeeping staff. Fourteen percent of the whole cohort had minimal knowledge of TB despite working in a hospital that treats MDR-TB daily. The overlap between the groups with the lowest knowledge and the groups with the highest disease rate is the practical message of this study: housekeeping staff simultaneously occupied the top of the risk ranking and the bottom of the knowledge ranking.

This overlap has been described before. Jelip et al. found that HCWs with TB disease in Sabah, Malaysia, were 5.9 times more likely to have poor knowledge about TB transmission and 4.3 times more likely to be unaware of the need for respiratory protection [27]. Luksamijarulkul et al. showed in Thailand that knowledge alone is insufficient: 97% of hospital personnel were knowledgeable about TB, yet only 52% used respirators, and only 72% implemented respiratory isolation for contagious cases [28]. Knowledge is necessary but not sufficient; in the groups at highest risk in our cohort, even that first condition is not met.

We found no difference in awareness scores between TB-affected and unaffected participants (median 15.0 in both, p = 0.804). This should not be read as evidence that knowledge is unrelated to risk. Awareness was measured after diagnosis in affected participants, several of whom had completed treatment and received counseling as part of NTEP care, so any pre-existing deficit may have been erased by the experience of the disease itself. A cross-sectional design cannot recover the knowledge state that preceded infection.

Health-related quality of life

The comparison of TB-affected HCWs with unaffected colleagues from the same workforce is, to our knowledge, not available elsewhere in the Indian HCW literature, and it is among the strongest evidence in this study. TB-affected HCWs scored lower in every SF-36 domain, significantly so in seven of eight, with an overall deficit of 24 points on a 100-point scale. The largest deficits were in role limitation due to physical health, general health perception, role limitation due to emotional problems, and social functioning. Because both groups work in the same institution, under the same conditions, the comparison controls informally for the occupational environment in a way that a single-group description cannot.

Two features deserve comment. First, the deficits in the two role-limitation domains and in social functioning are as large as those in physical functioning, indicating that the burden of TB in this workforce is substantially one of disrupted work and social participation rather than physical incapacity alone. Second, emotional well-being was the only domain not reaching significance, while role limitation due to emotional problems showed one of the largest deficits. This dissociation suggests that affected HCWs experience impaired functioning attributed to emotional causes without necessarily reporting worse mood on direct questioning, a pattern compatible with the stigma described by Abbas Ali et al. [16] and von Delft et al. [26].

These scores are also a reminder that a single-group description can mislead. A mean overall score close to 50 on a 0-100 scale invites the reading that quality of life in this group was broadly acceptable; measured against colleagues in the same hospital, it was not.

Complete SF-36 responses were available for 11 of the 15 affected HCWs, so non-response could bias the comparison. The groups were not matched on age, sex, or occupational category, each of which is associated with both TB status and quality of life in these data, and with 11 affected participants, no adjusted analysis was feasible. Finally, socioeconomic status was not captured, and TB-related stigma together with social desirability bias may have influenced self-reported responses in either direction.

Implications for practice

Our findings translate into four specific actions. First, institutional TB infection control plans should be written to cover the whole workforce, not the clinical workforce. WHO recommends that every facility designate a TB infection control officer, allocate a dedicated budget, provide N95 respirators, and mandate TB infection control training at least once every two years [29]; in our setting, the priority group for that training is housekeeping, security, technical, and clerical staff, and it must be delivered in the local language and at a literacy level appropriate to the audience. Second, the FAST strategy (Find cases Actively using cough screening and rapid molecular diagnostics, Separate temporarily, Treat effectively) offers a practical administrative framework that reduces the duration of unsuspected infectiousness on the wards [30]. Third, environmental controls including improved ventilation, reduced overcrowding, and germicidal ultraviolet air disinfection are essential in a facility with this MDR-TB throughput [31]. Fourth, periodic occupational screening should be offered to all staff categories and should incorporate LTBI testing by tuberculin skin test or interferon-gamma release assay, which our study did not perform and which would identify the reservoir from which future active cases will arise. Given the quality-of-life deficits we document, occupational health services should also provide structured psychosocial support and a defined return-to-work pathway for affected staff.

Limitations

This study has several limitations. We surveyed only currently employed staff, so HCWs who had left, transferred, retired, or died because of TB would not have been captured. TB status was also self-reported through the screening questionnaire and was not cross-checked against NTEP or hospital treatment records. Both of these are forms of survivorship and reporting bias that would tend to understate the burden, so the figures we report are more likely to be conservative than inflated. We did not test for LTBI, and the size of the infection reservoir in this workforce therefore remains unknown.

The number of staff approached and the institutional staffing establishment were not recorded, so we could not calculate a response rate or judge how closely the sample mirrors hospital staffing. Reporting rates within each occupational category, rather than the share of cases contributed by each, reduce but do not remove the effect of this. Smoking status, nutritional status, and use of PPE were not recorded at all; smoking and undernutrition are established risk factors for TB in India [5] and may confound the occupational differences we describe. The study was conducted at a single center that serves as the district TB center and carries an unusually high MDR-TB caseload, so the burden seen here may exceed that of hospitals without that role.

With 15 cases spread across 10 occupational categories, CIs are wide and multivariable adjustment was not possible, so the contributions of sex, age, and occupational group could not be separated. The sample size had been calculated for an expected prevalence of 30%, well above the 3.6% observed, and precision around the primary estimate is correspondingly lower than intended. Occupational category was derived from free-text responses after data collection, and awareness levels were assigned on review of the completed questionnaires rather than against a fixed score, although the occupational gradient was similar whether the assigned category or the item score was used. These findings should therefore be read as hypothesis-generating and need confirmation in a larger multicenter study that follows staff over time and includes those who leave employment.

## Conclusions

Among 250 HCWs at a tertiary care hospital serving as the district TB center for Raigad district, Maharashtra, the point prevalence of current active TB was 3.6% (95% CI 1.9-6.7), and 6.0% had current or recent TB, a burden approximately an order of magnitude above the national adult population prevalence of pulmonary TB. One in five affected HCWs had MDR-TB. Once occupational denominators were applied, risk was concentrated in non-clinical and support staff rather than in doctors (14.3% versus 4.3%; OR 3.69, 95% CI 1.24-10.99), and this same workforce demonstrated markedly poorer TB knowledge, with good awareness in 8.7% of housekeeping staff against 87.8% of doctors. Affected HCWs scored lower than unaffected colleagues from the same institution across all eight SF-36 domains, significantly so in seven, with an overall deficit of 24 points.

The practical implication is specific rather than general: TB infection control at facilities of this type should be reoriented so that administrative, environmental, and respiratory-protection measures, and mandatory recurrent training delivered in local languages, reach housekeeping, security, technical, and clerical staff with at least the same rigor currently applied to clinical staff. Periodic screening of all staff categories incorporating LTBI testing, and structured psychosocial and return-to-work support for affected staff, should accompany these measures. Because our occupational findings rest on a small number of events at a single institution, they require confirmation in a multicenter cohort with denominators adequate to estimate category-specific risk directly.

## Appendices

Appendix A 

**Table 7:** Questionnaire 1: tuberculosis screening questionnaire.

1.			Circle any of the below symptoms you have today													
			Cough lasting longer than 2 weeks												
			Coughing up blood (hemoptysis)												
			Fever												
			Unexplained weight loss												
			Night sweats												
			Loss of appetite for more than a month												
			Any palpable swelling in the neck, groin, or the axilla												
			Abdominal pain and altered bowel habits												
			Seizure and headache												
2.			Are you suffering from HIV/AIDS? Yes/no													
3.			Are you a diabetic for more than a year? Yes/no													
4.			Are you on treatment with any immunosuppressants for more than 3 months? Yes/No													
(A) Are you a patient with tuberculosis/have you ever had a positive TB skin test or TB blood test? Yes/no																
(If yes, kindly answer the following questions)																
(If no, kindly move to section B)																
Circle the site of TB: Lungs/lymph nodes/liver/abdomen/brain/uterus/ovary/others. If others, kindly mention: _____																
Are you taking anti-tuberculosis therapy (ATT) for DOTS?							Yes			No			I don’t know			
Do you have any side effects from the medication?							Yes			No			I don’t know			
Have you developed yellowing of the eyes, abdominal pain, severe vomiting, or melena after taking the medicines (deranged liver enzymes + symptoms of hepatitis)							Yes			No			I don’t know			
Have you had joint pains after taking the medicines?							Yes			No			I don’t know			
Do you suffer from gastritis after taking the medicines?							Yes			No			I don’t know			
Have you had any complaints of peripheral neuropathy (tingling sensations and burning pain in extremities) while on treatment?							Yes			No			I don’t know			
Did you have blurring of vision during treatment?							Yes			No			I don’t know			
Do you have other side effects you want to mention? If yes, kindly mention below							Yes			No			I don’t know			
Other side effects: _________																
Have you faced any difficulties getting diagnosis and treatment of TB?			Yes				No				Somewhat					
Have you undergone any mental trauma/depression during treatment?			Yes				No				I don’t know					
Has your family been supportive during treatment?			Yes				No				Somewhat					
Have the healthcare staff who treat you (doctors, nurses, DOTS personnel) been kind and empathetic?			Yes				No				Somewhat					
Were you counseled about the treatment, side effects, and outcome before you were given treatment?			Yes				No				Somewhat					
Are you getting treatment regularly?			Yes				No				I don’t know					
Have you taken BCG vaccine?			Yes				No				I don’t know					
How were you diagnosed? (tick appropriate)			(A) Sputum examination		(B) Lymph node		(C) CSF		(D) Biopsy for abdomen				(E) Biopsy for bone		(F) Bronchoscopy	
(B) Have you had TB in the past? Yes/no (If yes, kindly answer the following questions)																
Circle the site of TB: Lungs/lymph nodes/liver/abdomen/brain/uterus/ovary/others. If others, kindly mention: _____																
Did you have any side effects from the medication?			Yes				No				I don’t know					
Did you develop yellowing of eyes, abdominal pain, severe vomiting, or melena after taking the medicines (deranged liver enzymes + symptoms of hepatitis)			Yes				No				I don’t know					
Did you get joint pains after taking the medicines?			Yes				No				I don’t know					
Did you suffer from gastritis after taking the medicines?			Yes				No				I don’t know					
Have you had any complaints of peripheral neuropathy (tingling sensations and burning pain in extremities) while on treatment?			Yes				No				I don’t know					
Did you have blurring of vision during treatment?			Yes				No				I don’t know					
Did you have other side effects you want to mention? If yes, kindly mention below			Yes				No				I don’t know					
Other side effects: _________																
Did you face any difficulties getting a diagnosis and treatment of TB?		Yes				No								Somewhat		
Did you undergo any mental trauma/depression during treatment?		Yes				No								I don’t know		
Was your family supportive during treatment?		Yes				No								Somewhat		
Were the healthcare staff who treated you (doctors, nurses, DOTS personnel) kind and empathetic?		Yes				No								Somewhat		
Were you counseled about the treatment, side effects, and outcome before you were given treatment?		Yes				No								Somewhat		
Did you get your treatment regularly?		Yes				No								I don’t know		
Did you feel embarrassed about having TB?		Yes				No								Somewhat		
Did you get emotional and social support from your colleagues and peers?		Yes				No								Somewhat		
Did you feel embarrassed about having TB?		Yes				No								Somewhat		
Did you face any social stigma/inequality while being an active TB patient?		Yes				No								Somewhat		
Do you still face social stigma/inequality?		Yes				No								Somewhat		
How were you diagnosed? (tick appropriate)	(A) Sputum examination			(B) Lymph node	(C) CSF			(D) Biopsy for abdomen				(E) Biopsy for bone			(F) Bronchoscopy	

Appendix B 

**Table 8:** Questionnaire 2: awareness assessment questionnaire.

Do you know what tuberculosis is?	Yes	No	Somewhat
If so, do you know what its etiology is?	Yes	No	Somewhat
Do you know how TB spreads?	Yes	No	Somewhat
Do you know the common symptoms of TB?	Yes	No	Somewhat
Are you aware that there is free treatment for TB under a government program?	Yes	No	Somewhat
Have you heard of DOTS/NTEP?	Yes	No	Somewhat
Do you know about different types of TB like drug-sensitive TB, drug-resistant TB, multidrug-resistant TB, extensively drug-resistant TB?	Yes	No	Somewhat
Do you know TB can be cured?	Yes	No	I think so
Do you know the names of anti-tuberculosis drugs used to treat drug-sensitive TB?	Yes	No	Somewhat
Do you know the names of anti-tuberculosis drugs used to treat drug-resistant TB?	Yes	No	Somewhat
Do you know which doctor/department to approach for TB screening/treatment?	Yes	No	Somewhat
Do you know the duration of treatment needed to cure drug-sensitive TB?	Yes	No	Somewhat
Do you know the duration of treatment needed to cure drug-resistant TB?	Yes	No	Somewhat
Are you aware that anti-TB drugs can have side effects?	Yes	No	Somewhat
Do you know how TB is diagnosed?	Yes	No	Somewhat
Have you heard of the test GeneXpert (CBNAAT)?	Yes	No	Somewhat
